# Air Quality Monitoring for Vulnerable Groups in Residential Environments Using a Multiple Hazard Gas Detector

**DOI:** 10.3390/s19020362

**Published:** 2019-01-17

**Authors:** Yujiao Wu, Taoping Liu, Sai Ho Ling, Jan Szymanski, Wentian Zhang, Steven Weidong Su

**Affiliations:** 1 Centre for Health Technologies, School of Biomedical Engineering, Faculty of Engineering and Information Technology, University of Technology Sydney, Sydney, NSW 2007, Australia; yujiaowu111@gmail.com (Y.W.); taoping.liu@student.uts.edu.au (T.L.); Steve.Ling@uts.edu.au (S.H.L.); wentian.zhang@student.uts.edu.au (W.Z.); 2 School of Electrical and Data Engineering, Faculty of Engineering and Information Technology, University of Technology Sydney, Sydney, NSW 2007, Australia; Jan.Szymanski@uts.edu.au

**Keywords:** electronic nose, environmental monitoring, remote sensing and control

## Abstract

This paper presents a smart “e-nose” device to monitor indoor hazardous air. Indoor hazardous odor is a threat for seniors, infants, children, pregnant women, disabled residents, and patients. To overcome the limitations of using existing non-intelligent, slow-responding, deficient gas sensors, we propose a novel artificial-intelligent-based multiple hazard gas detector (MHGD) system that is mounted on a motor vehicle-based robot which can be remotely controlled. First, we optimized the sensor array for the classification of three hazardous gases, including cigarette smoke, inflammable ethanol, and off-flavor from spoiled food, using an e-nose with a mixing chamber. The mixing chamber can prevent the impact of environmental changes. We compared the classification results of all combinations of sensors, and selected the one with the highest accuracy (98.88%) as the optimal sensor array for the MHGD. The optimal sensor array was then mounted on the MHGD to detect and classify the target gases without a mixing chamber but in a controlled environment. Finally, we tested the MHGD under these conditions, and achieved an acceptable accuracy (70.00%).

## 1. Introduction

The indoor environment plays an important role in an individuals overall health. The occurrence of a new class of diseases, identified as building-related illnesses (BRI) and sick building syndromes (SBS), arising from the long-term occupancy in confined living spaces, such as office buildings or homes, and being caused by chemical contaminants, in particular volatile organic compounds (VOCs), has motivated recent interest in indoor air quality (IAQ) monitoring [1]. Studies have linked IAQ to mental health and other illnesses that are not easily noticeable in the short-term but can be major concerns in the long-term [2]. Typically, we can find thousands of indoor chemical contaminants, including by-products of combustion (Nitrogen dioxide (${\mathrm{NO}}_{2}$), Sulfur dioxide (${\mathrm{SO}}_{2}$), carbon monoxide (CO), etc.), cigarette smoke, particulate matter, rotten meat, mineral fibers, and a number of volatile organic compounds. In spite of the very low concentrations, some of these compounds are extremely toxic, like ${\mathrm{NO}}_{2}$ or CO; some others, like benzene and formaldehyde, were proved to be carcinogenic. Therefore, the monitoring of air quality is of paramount importance to ensure safe living conditions.

In the last few years, the electronic nose (popularly known as the e-nose) has been widely applied to gas detection/identification in many real-world applications as a general gas detector. For example, the e-nose has been utilized in combustion processes, e.g., rocket combustion and forest fire smoke [3,4]. In environmental monitoring processes, the e-nose has been applied to analyze the principal atmospheric pollutants emitted from combustion processes, which contain carbon monoxide and dust [5]. Furthermore, the e-nose can be used for food quality assessment [6,7] by determining the amount of off-odor food in packaging materials, for medical diagnostics [8,9], as well as for monitoring the harmful gas species related to health and safety issues in the home.

The objective of this project is to develop a robot (a multiple hazard gas detector (MHGD)) using e-nose technology to help vulnerable people by detecting harmful gases more quickly and precisely.

For example, the detection of off-odor rotten meats prevents the misuse of spoiled meat which may lead to severe diarrhea, amoebiasis, and life-threatening intestinal infections in aging people. The detection of inflammable gases, such as ethanol, aims to provide an early warning system for the safety of elderly people. The MHGD notices the hazardous gas after taking samples, and then gives an early warning. This is especially important for those who suffer olfactory impairment or movement disorders and so cannot smell or find the potential fire.

Furthermore, homes with little kids, pregnant women, and patients with breathing problems also need such safety precautions. Exposure to secondhand smoke causes numerous health problems in infants and children, including more frequent and severe asthma attacks, respiratory infections, ear infections, and sudden infant death syndrome (SIDS) [10]. It has been shown that smoking during pregnancy results in more than 1000 infant deaths in the USA annually [11]. Thus, it is essential to produce a real-time detector to protect the infant and the pregnant woman from hazardous materials and gases. A hazardous gas detector can act to reassure these people, lessening their potential exposure to risks, and thus improving their quality of life.

Traditionally, ceiling-fixed sensors can have a delay when the origin of the smoke is far away, even failing to detect the hazardous smoke altogether because of the low concentration of the gas. The designed MHGD can be setup to be much more sensitive and responsive in detecting the “smell” of the smoke than traditional fixed sensors. Additionally, the MHGD can provide powerful self-designed models, such as defining an alarm grading system, to only alert customers themselves without disturbing the neighbors.

However, the generated data from the e-nose may contain irrelevant information, and moreover, the principles of the new field of research can be very complex, some of them never having been studied before. Thus, machine learning as a powerful tool for advanced data processing has become a core technique for e-nose development. A complete machine-learning process consists of data preprocessing, feature extraction and dimension reduction, and system modeling [12]. The sensor signals are composed of a large number of variables, after the data pre-processing, feature extracting methods are used to transfer signals from original high-dimensional space to a low-dimensional feature space or to select “representative” (pertinent) variables to characterize the whole system. Furthermore, machine learning is utilized to establish models for classification.

In the sensor optimization, we compared three machine learning methods: support vector machine algorithms (SVMs), k-nearest neighbors algorithm (kNN), multinomial logistic regression (also known as Softmax regression). The experimental results show that the proposed MHGD system can detect the different gases quickly and classify the odors accurately. Furthermore, considering different applications, we provide three strategies for customers to choose from.

In this study, the contribution of the designed system is reflected in four major parts. First, we describe the construction a four-wheel tracing car, through the open-source platform, that can automatically track trace and avoid obstacles based on ultrasonic sensors. It is also equipped with an e-nose system and an information feedback alarm system to perform automatic tracing indoors so as to identify where the potential danger source is, especially for gas-generated hazardous events. Second, we describe the loading of the e-nose on a four-wheel tracing car, making it able to detect ambient gas composition and classify any detected gas using a machine learning algorithm to decide whether to send an alarm or not. Third, we describe how through a Wi-Fi signal transmission module and buzzer alarm device, it can achieve closed-loop feedback on the collected information and use this in order to alert the customer of the danger and call for help. Fourth, we present an exhaustive strategy to show the trade-off between the cost of whole system and classification accuracy.

The remainder of the paper is organized as follows. The next section presents the proposed architecture of the whole system. In Section 3, we introduce the machine learning and data analysis method we use. In Section 4, we present the data processing, gas classification, and its associated experimental results. Section 5 presents the analysis and discussion based on the experiment results. Finally, Section 6 concludes the paper.

## 2. Gas Detection Platform

**Figure 1:**
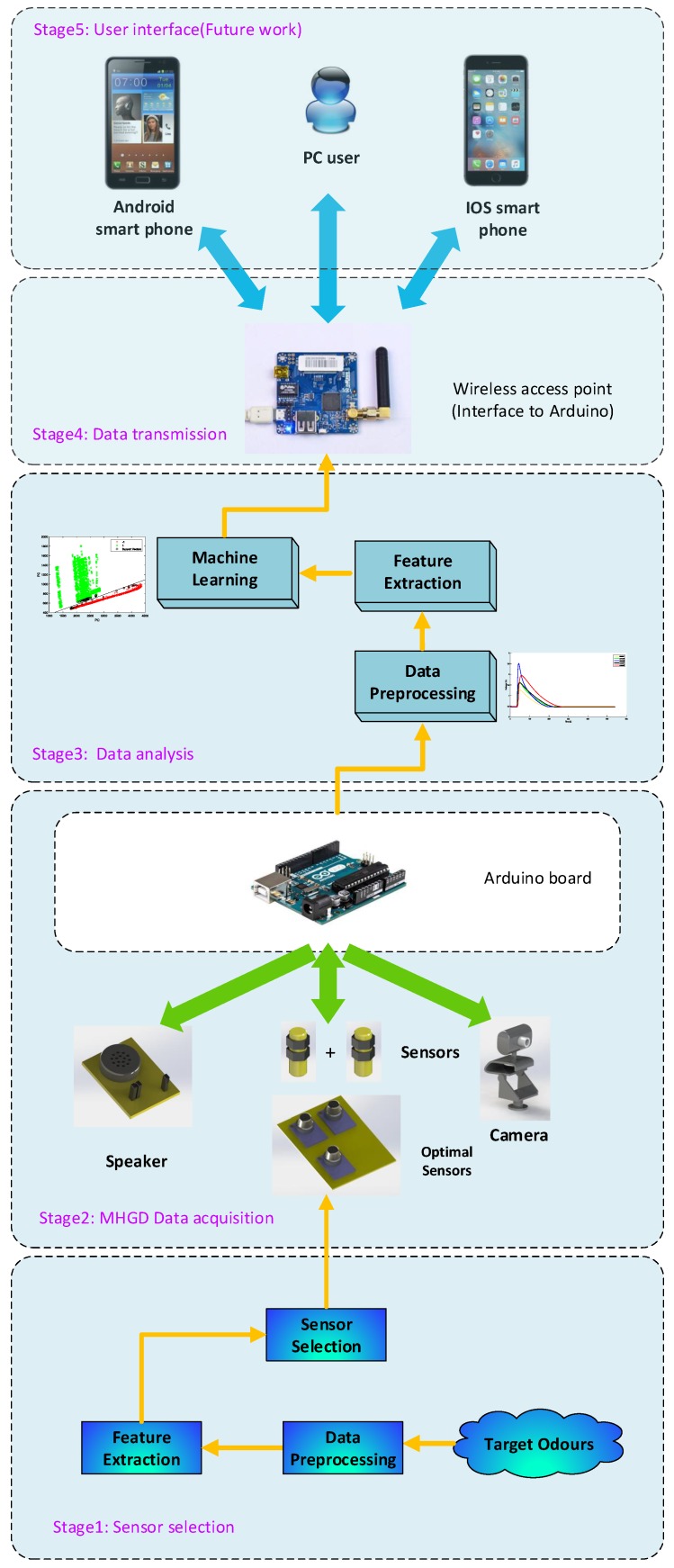
An overview of the proposed system.

**Figure 2:**
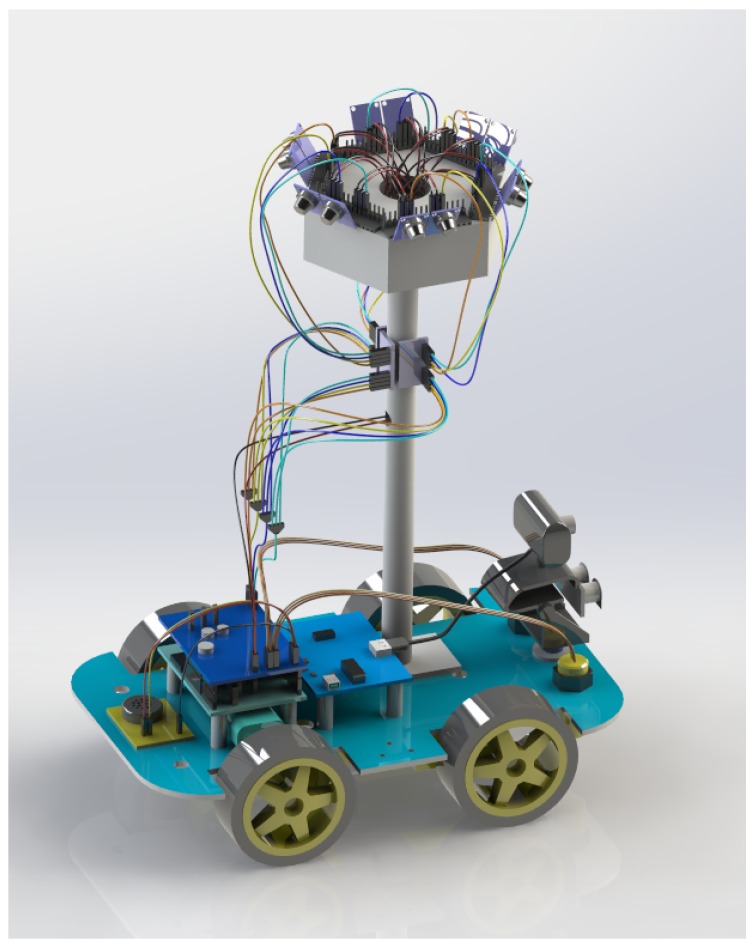
Model of multiple hazard gas detector (MHGD).

In order to address the aforementioned issues and the potential hazardous events, we designed and implemented a novel, flexible, and portable domestic hazardous odor detection system using an e-nose system, with a small net that consists of micro-controller board Ethernet Shield, hardware interface modules and the iOS, Android smartphone App, and PC Software. An overview of the proposed detector architecture is shown in Figure 1. The model of the proposed detector is shown in Figure 2.

As shown in Figure 1, the whole system consists of four parts: Stage 1 is sensor optimization, Stage 2 goes through the processes of gathering data with the MHGD, Stage 3 is intended as a data processing step, working through what methods are used, how the features are gathered, and what tools are used, Stages 4 and 5 detail the process of how the data is transmitted to the user interface.

In first stage, we applied a gas sampling device to do the data gathering work by collecting the dataset in a controlled environment. Those data were used to test the sensors responses regarding different odors and to verify the feasibility of our hypothesis.

Moving on from the sensor reduction stage, the next stage is intended to briefly show the hardware components of the MHGD. Using the optimized sensor array, we developed a MHGD with a camera, speaker, ultrasonic sensors, infrared sensors, and Micro Controller Unit (MCU) mounted on a vehicle-based robot that can be remotely controlled.

The third stage shows the data processing steps. The real data can contain a lot of noise and so must go through three stages: The first step is data preprocessing, which eliminates the noise from the real data. The data may still contain information that we do not need, so the next step is feature extraction, which aims to generate data that contains as much information as possible to present the original data. The last step is intended to classify the specific gas using machine learning.

The fourth stage is the data transmission stage, where the classification result is transmitted to the user interface through a wireless access point.

### 2.1. MHGD System Hardware Development

Mounted on a four-wheel car, this automated hazard gas detection system consists of three main hardware components, including the main board, the customer interface, and the e-nose system. The main hardware is composed of a simple, open hardware design for the Arduino board with an Atmel AVR processor and onboard I/O support, while the software side of the Arduino consists of a standard programming language and a boot-loader which runs on the board. One of the main advantages of the MGHD is its flexibility for mobile olfaction tasks. The mechanical design of the MHGD is open to a variety of possible configurations. It can work either as a conventional home gas detector, smelling for any possible hazardous gases, or as a mixed hazardous gas detector by replacing different combinations of sensors on the specially designed socket, which uses bionics on the top of the device to focus on detecting and classifying specific gases. In our system, the hardware interface modules are directly connected with every single other module with wires. In order to make our system more scalable, we use ADS1015 (Texas Instruments Inc., Dallas, TX, USA) to connect the main board with the e-nose system. The ADS1015 device incorporates a low-drift voltage reference and an oscillator, a programmable gain amplifier (PGA), and a digital comparator. These features, along with the wide operating supply range, make the ADS1015 well suited for our system, which is a power and space-constrained, sensor measurement application. In the proposed system, we can use three ADS1015 chips, so our system can have up to 12 different gas sensors, as shown in Figure 2.

### 2.2. Software Development

The system shown in Stage 4 and Stage 5 consists of an application developed using the Android and iOS platform and a micro-controller board Ethernet-based wireless access point. The open-source micro-controller is the main controller that hosts the micro web-server and performs the necessary actions. The sensors and actuators (motors) are directly interfaced to the OS controller. The multiple hazard gas detector can be controlled and monitored from a remote location using the smart home application, which will communicate with the micro web-server via the wireless access point.

In Stage 5, the data from wireless access point is transmitted to the users devices through an Internet connection via Wi-Fi. Currently, we only have a simple user interface. However, in the future, we will design a real-time home hazardous gas interface which can be installed on three different software platforms for users. The present, simple smartphone application, as well as the PC software, provides a graphical user interface (GUI) for accessing and controlling the device. The iOS application is created by the X-code. Most of the programming was implemented using Cocoa Touch and Objective-C code. For the Android App, Android Studio or Eclipse was used to create the application. The PC application was based on the .NET 4.5 framework. Visual Studio was used to create the software. Then, we created a solution .SLN file. The main project consisted of four parts, including the main interface initialization, system settings, video stream settings, and servo angle settings.

## 3. Data Analysis

The sampling signals are analyzed for the benefit of fast and robust recognition in our application. The data analysis process is composed of three parts: data preprocessing, feature extraction, and pattern recognition. The signals obtained from our device normally contain noise, data preprocessing is used to eliminate this noise, and then features with certain geometric definitions were utilized to represent the entire signal. The set of parameters served as an input to a classification process for sample identification.

### 3.1. Signal Pre-Processing

Signal preprocessing is an essential element in e-nose instruments. In order to increase the signal-to-noise ratio (S/N) and reduce the sensor drift, the analysis of the first raw sensor signals originating from the chemical sensor array requires a data preprocessing stage. Herein, we applied a median filter, mean filter, and normalization in data preprocessing. The formula for the fractional conductance in this study is as follows:

$$x_{i}=\frac{x_{max}-x_{min}}{x_{min}}$$

where $x_{i}$ is the response value of a certain point in the response curve, $x_{max}$ and $x_{min}$ represent the maximum and minimum value, respectively.

Time parameters of 1st-order and 2nd-order derivatives are sensitive to noise. In order to eliminate noise and improve the accuracy, a mean filter and medium filter were adopted in this paper. A median filter was used to remove the outliers of the data. After that, the curve became stepped, so the mean filter was adopted to smooth out the result of median filter. Then, fractional conductance was adopted to reduce the effect of sensor drift.

### 3.2. Feature Generation

**Figure 3:**
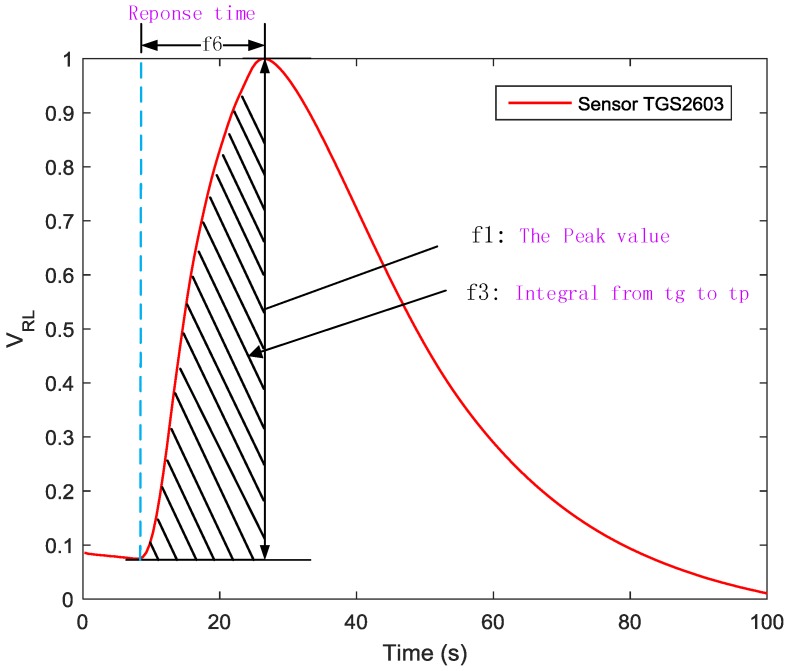
Feature 1, 3, and 6 extracted from each sensor response curve.

**Figure 4:**
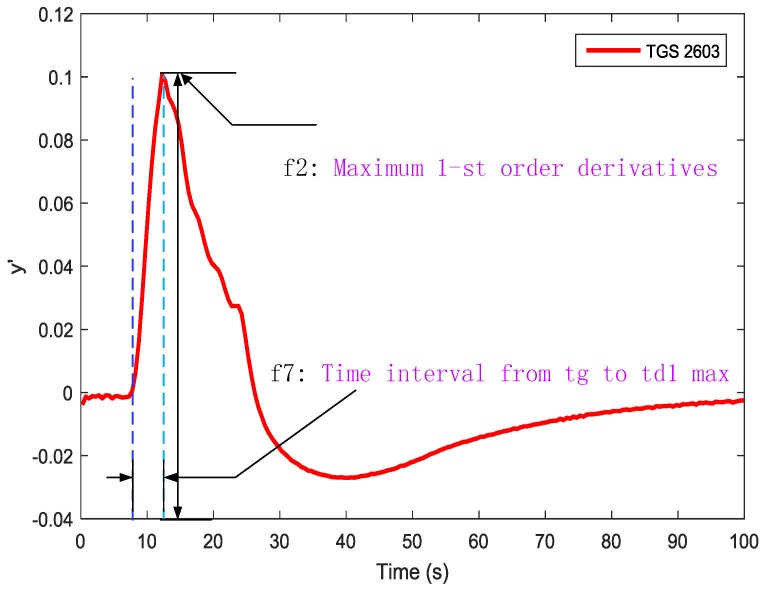
Feature 2 and 7 extracted from the 1st-order derivative-based sensor response curves.

**Figure 5:**
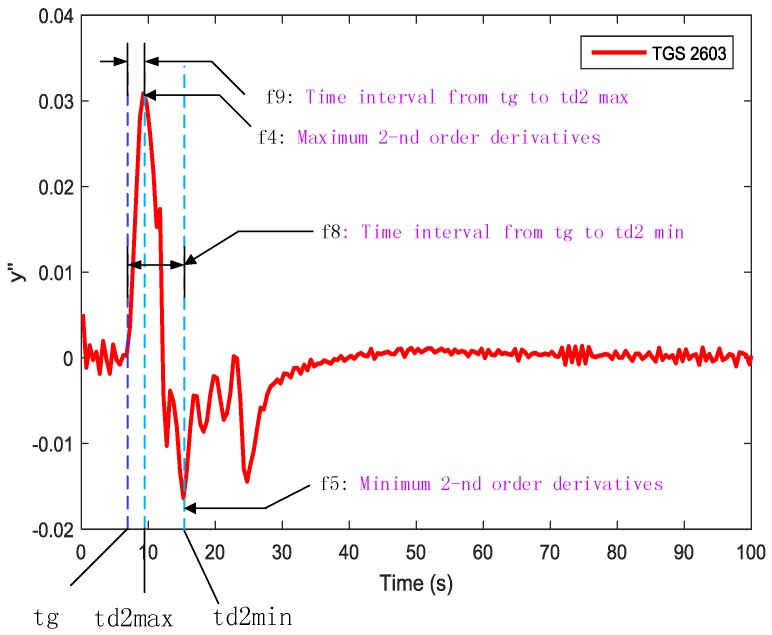
Feature 4, 5, 8, and 9 extracted from 2nd-order derivative-based sensor response curves.

‘Gas prints’ collected from the e-nose were converted into electrical signals, which is more suitable for data analysis. However, as result of the properties and limitations of current sensors, this can cause the distortion of the available information. In order to reflect different information related to the reaction kinetics at different phases [13] and obtain much more information from the multivariate time responses of the sensor arrays, we applied the traditional feature extraction method [14,15]. Integrals, differences, primary derivatives and secondary derivatives at a certain interval (as shown in Table 1) from the response curves were extracted as shown in Figure 3, Figure 4 and Figure 5.

Those features were chosen according to the characteristics of the sensor response curves. We extracted nine features from each sample, as is shown in Table 1.

### 3.3. Machine Learning Techniques

The last step in the data analysis is to establish models for classification. The features extracted were served into subsequent classification tasks. Three kinds of classification algorithms were applied for target gas detection and used to process the recorded data for identifying gas mixture components and a contamination estimation. In this section, we illustrate the basic principles and some important details of the three selected algorithms, which include k-nearest neighbors (kNN), support vector machine (SVM), and Softmax regression.

#### 3.3.1. The k-Nearest Neighbors

The kNN is a powerful technique that can be used to generate highly nonlinear classifications with limited data [16]. To classify an example, the kNN finds the closest examples in the dataset and selects the predominant class among these neighbors. The kNN can generate highly local decision regions by choosing an appropriate value to present very attractive asymptotic properties: as the number of examples approaches infinity, the probability of error for the (K = 1) NN classifier will not be worse than twice the Bayes error, the best any classifier can achieve [17].

#### 3.3.2. Support Vector Machines

SVM is a popular machine learning method for classification, regression, and other learning tasks.The objective of SVM is to find a hyperplane with the maximum margin to separate positive and negative samples [18]. Considering that, any hyperplane is in the form of Equation (1):

(1) $$\omega^{T}x+b=0$$

where ω and *b* represent the weight and bias, respectively. Then, we need to optimize the values of ω and *b* to maximize the distance between the two different samples.

#### 3.3.3. Softmax Regression

Softmax regression [19], also known as multinomial logistic regression, is a generalization of logistic regression to tackle multi-class classification problems. As Softmax regression is a multi-class classifier, thus, the desired labels for Softmax regression is defined as $y^{\left(i\right)}\in \left\{0,1,\ldots ,K\right\}$, k=1,…,K where *K* is the number of classes and i=1,…,M, where *M* is the number of training set.

In the Softmax regression model, the function of mapping the original data of the input layer to the different class units of the output layer is the discriminant function $h_{\theta }\left(x\right)$ of the model and defined as

(2) $$h_{\theta }\left(x\right)=\left[\begin{array}{c} P(y=1|x;\theta )\\ P(y=2|x;\theta )\\ \vdots \\ P(y=K|x;\theta ) \end{array}\right]=\frac{1}{\sum_{j=1}^{K}\exp\left(\theta^{(j)T}x\right)}\left[\begin{array}{c} \exp(\theta^{(1)T}x)\\ \exp(\theta^{(2)T}x)\\ \vdots \\ \exp(\theta^{(K)T}x) \end{array}\right]$$

where $\theta^{\left(1\right)},\theta^{\left(2\right)}\ldots ,\theta^{\left(K\right)}$ are the parameters of Softmax regression model.

## 4. Experiment and Analysis

To ensure a low-cost and efficiency of the whole system, the experimental system was designed in two parts: sensor array optimization in a closed environment and target odor detection in an open environment. In the first step, sensor optimization was carried out on a stationary e-nose device with a sealed sensor chamber. The aim of this was to test whether the selected sensors are capable of changing their behavior when exposed to volatile substances released by three selected analytes.

The samples used in the experiment were three hazardous gases: the odor from spoiled rotten meat, the gas ethanol, and smoke from a burning cigarette, this was chosen particularly as emissions from cigarette tobacco comprise a wide range of chemical components making up a complex odor [20]. Real samples were used in our experiments. In all experiments, the weight of spoiled meat was 4 g. The volume of liquid ethanol was 2 mL. 0.3 g burning tobacco in a headspace bottle served as sample of cigarette smoke. The odor was injected into the sealed e-nose chamber with a sampling needle. After that, clean air was pumped into chamber. The sensor array used in the gas recognition experiments was composed of three elaborately selected gas sensors: TGS2620, TGS2603, and TGS2600 (Figaro Engineering Inc., Osaka, Japan). The response characteristics of these sensors are listed in Table 2. Sampling was done in three phases: the baseline phase, the sampling phase, and the recovery phase. Each test lasted for 50 s. All the measurement data were stored on computer for future processing and analysis. The gases were collected in a closed, controlled environment with an ambient temperature of 25–27 degree Celsius and 50% RH ambient humidity. The experiment for each target odor was carried out 50 times. We eventually acquired 150 samples in total.

In the second step, the optimized sensor array was installed on the MHGD to detect target odors. Since the sensor array was directly exposed to the room environment, the conditions are referred to as an “open environment”. According to a previous study [21], the features mentioned in Table 1 have an influence when in moving conditions (i.e., moving sensing device or moving gas source). Therefore, all gases were collected in a closed, controlled environment, that is, no-wind and stationary platform conditions. All experiment were carried out at room temperature. In this way, the testing system had to remove the interference from the atmosphere, so that the repeatability of the response curves collected by MHGD was guaranteed. Ethanol gas and off-flavor rotten meat were adopted in this experiment. For sample delivery, if we take the cigarette smoke as an example, the burning tobacco in a headspace bottle was served as the sample. Before testing, the distance between the headspace bottle with burning tobacco and MHGD was 15 cm. Testing was repeated 10 times for each sample. Each test lasted for 300 s. Eventually, we obtained 30 group data sets in total.

### Data Analysis

**Figure 6:**
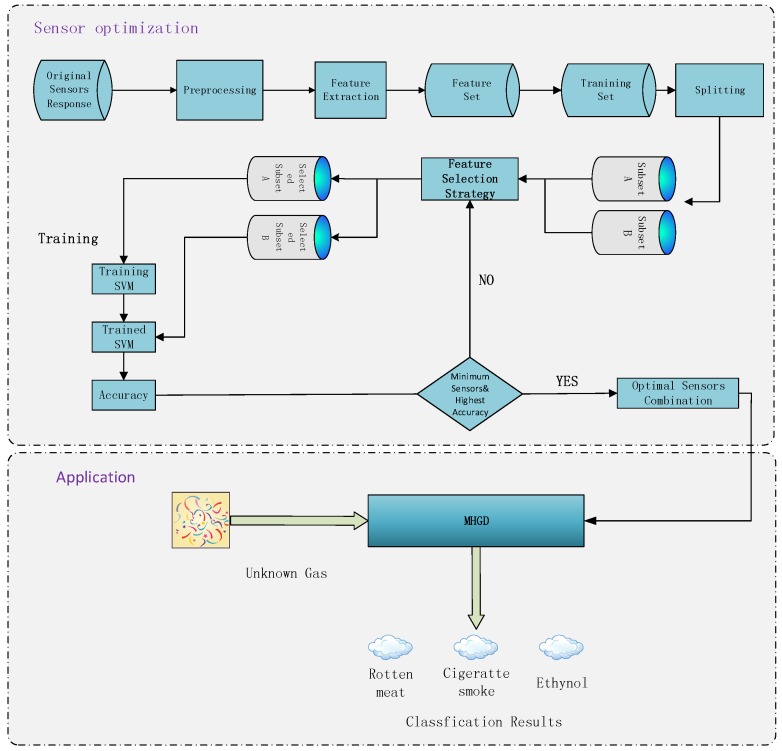
The data flow diagram of MHGD.

**Figure 7:**
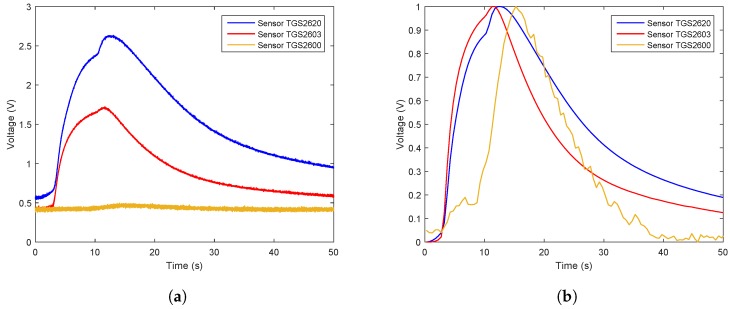
Time series of ethanol raw data and the data after preprocessing. (**a**) Shows the representative example of the time series of the original response curves of the sensor array for gas ethanol. (**b**) Shows the results after preprocessing.

**Figure 8:**
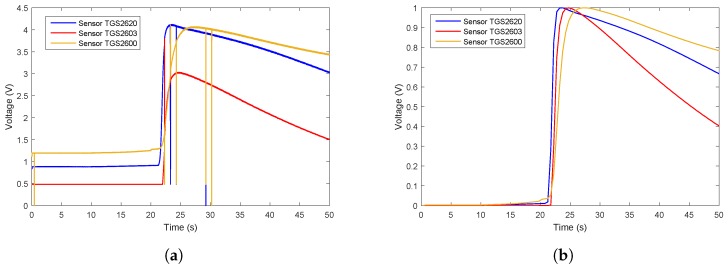
Time series of rotten meat odor raw data and the data after preprocessing. (**a**) Shows the representative example of the time series of the original response curves of the sensor array for the odor from rotten meat. (**b**) Shows the results after preprocessing.

**Figure 9:**
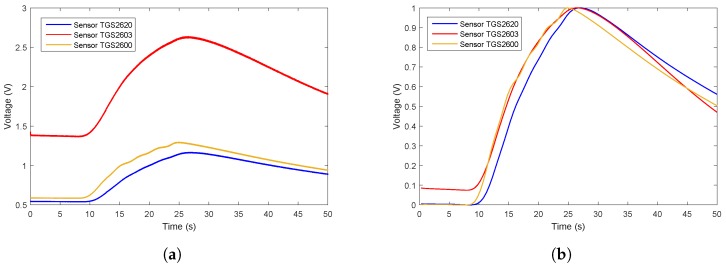
Time series of smoking gases raw data and the data after preprocessing. (**a**) Shows the representative example of the time series of the original response curves of the sensor array for gases from burning cigarettes. (**b**) Shows the results after preprocessing.

**Figure 10:**
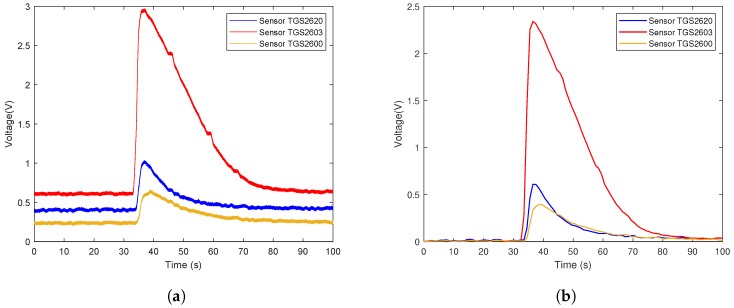
Time series of ethanol raw data and the data after preprocessing in the second experiment. (**a**) Shows the representative example of the time series of the original response curves of the sensor array for gas ethanol. (**b**) Shows the results after preprocessing.

**Figure 11:**
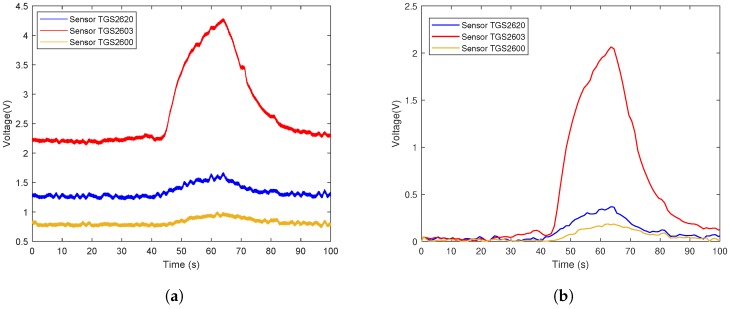
Time series of rotten meat odor raw data and the data after preprocessing in the second experiment. (**a**) Shows the representative example of the time series of the original response curves of the sensor array for the odor from rotten meat. (**b**) Shows results after preprocessing.

**Figure 12:**
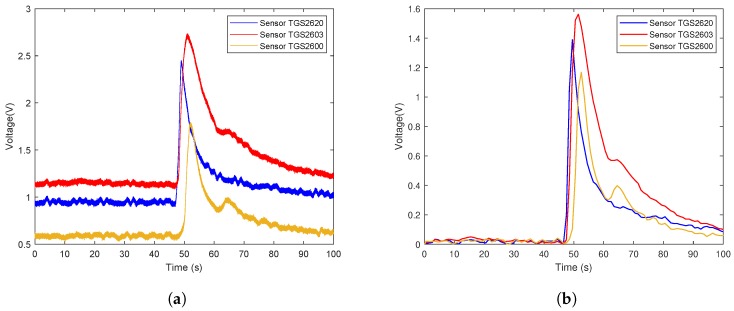
Time series of smoking gases raw data and the data after preprocessing in the second experiment. (**a**) Shows the representative example of the time series of the original response curves of the sensor array for gases from burning cigarettes. (**b**) Shows the results after preprocessing.

The data flow diagram is presented in Figure 6. Considering that the trend of sensor array curves collected from the same gas is similar, only one measurement of each gas is selected to illustrate the original data and the effect of data preprocessing. The response curves are shown in Figure 7, Figure 8 and Figure 9. Individually, Figure 7a, Figure 8a and Figure 9a show the raw response curve of the sensor array and the Figure 7b, Figure 8b and Figure 9b present the curves after filtering and applying fractional conductance. To better illustrate the variability and problematics that can appear in a real scenario, the response curves in the second experiment are shown in Figure 10a, Figure 11a and Figure 12a.

Random shuffling of datasets was performed on Matlab. After this, each dataset was divided into two equal subsets before training. For 27 features from three gases, we applied three classification schemes. A two-fold cross validation (2-CV) method (75 samples for training and 75 for testing) was repeated for 300 times. Of the two subsets, one subset was retained as the testing set, and the other subset became of the training set. The entire dataset was used for both training and testing, which ensured that each sample was used for validation. The confusion matrix was calculated 300 times for the evaluation of three classification schemes and seven sensor combinations as is shown in the confusion matrix of the results in Table 3 and Table 4.

After data preprocessing, we applied three classifiers, SVM, kNN, and Softmax regression, to analyze the data. There were three sensors in the original sensor array. In order to balance the number of sensors and the classification accuracy, we trained three classifiers for all seven sensor combinations and found the best parameters for each classifier.

Table 3 shows the confusion matrix of the three single sensors. Table 4 shows the confusion matrix of the sensors array combination.

## 5. Results Analysis and Discussion

In this study, three classifiers were trained for different sensor combinations. First, the feature set generated from the sensor array from the e-nose system were provided to the algorithms as input vectors. Then, the train process of SVM, kNN, and Softmax regression were executed automatically using Matlab. Herein, we compare three strategies for customers to choose from.

In order to reduce the cost of the system, we trained the data from single sensors separately and found the best parameter for each classifier. As is shown in Table 3, for TGS2620, SVM has the highest sensitivity and specificity: 98.67% and 99.34%, respectively. For TGS2603, kNN has the highest sensitivity (96.00%) and specificity (98.00%). As for TGS2600, the highest sensitivity was achieved by SVM (98.88%) and the highest specificity (99.44%). Thus, in terms of the low-cost option, using TGS2600 with SVM gives an excellent performance.

The result of classification accuracy with all sensors with three classifiers is shown in Table 4. It was observed that all classifiers exhibit an excellent performance. The best performance was achieved by kNN at 99.33% with a specificity of 99.66%. This means that any classifier can achieve a high accuracy and there is no need to implement different classifiers to find the one with highest classification under the circumstances.

The result of classification accuracy with two sensors with all classifiers is also shown in Table 4. Under these circumstances, the highest classification accuracy was achieved by SVM with the sensor TGS2603 and TGS2600, which was better than a single sensor, the cost of the entire system also being lower than that of all three sensors. Thus, in terms of universal application, TGS2603 and TGS2600 show the best results.

SVM outperformed other classifiers in the first experiment. Therefore, we adopted SVM as the classification model in the second experiment. As a result of the limited number of samples, we applied a Leave-One-Out (LOO) strategy to train and test the classification model. The experiment of the MHGD in the open environment achieved an acceptable result, which reached an accuracy of 70% (21/30). The confusion matrix is shown in Table 5.

## 6. Conclusions

By integrating mobile devices, wireless communication, data acquisition and analysis systems, we designed and validated a multi-functional hazard gas detection system. It allows the users to detect various gases using mobile devices. Using this system as a framework, the design can be extended to various other applications, such as home air quality monitoring, fire detection, as well as hazardous gas detection in a chemical plant. To demonstrate the feasibility and effectiveness of the proposed solution, the system was tested for its ability to sense and classify the source of hazardous gases in a laboratory environment. The experimental results show that the proposed odor classification algorithm achieved the desired classification sensitivity and specificity.

The proposed system can be used to enhance the current air quality monitoring systems in residential buildings in terms of high classification accuracy, ease of deployment and integration into existing security and safety systems.

Future work will include the addition of extra features, such as seamless integration into existing infrastructures, development of a more intelligent user interface, and the improvement of the robustness of system. Furthermore, we will focus on performing sensing tasks in more sophisticated environments, taking different combinations of features into consideration to find the feature sets with the best and most reproducible performance. Simplification of the hardware, reduction in the power consumption, miniaturization and integration into the Internet of Things will also be considered.

## Figures and Tables

**Table 1:** Feature types and descriptions used in gas classification.

Type	Features	Description
Response based features	f1	The maximum response value
Derivative-based features	f2	Maximum 1st-order derivatives
Derivative-based features	f4	Maximum 2nd-order derivatives
Derivative-based features	f5	Minimum 2nd-order derivatives
Time parameter	f6	response time
Time parameter	f7	Time interval from tg to td1 max
Time parameter	f9	Time interval from tg to td2 max
Time parameter	f8	Time interval from tg to td2 min
Integral	f3	Integral from gas-on to peak

**Table 2:** Gas sensors sensitivity characteristics ^a^.

Sensors	Gas Type	Type	Producer
TGS2620	Ethanol, Hydrogen, Iso-butane, Carbon monoxide, Methane, etc.	MOS ^a^	Figaro (Japan)
TGS2603	Trimethylamine, Methyl mercaptan, etc.	MOS	Figaro (Japan)
TGS2600	Methane, Carbon monoxide, Iso-butane, Ethanol, Hydrogen, etc.	MOS	Figaro (Japan)

^a^ The response of sensors is non-specific, they are also sensitive to other gases, which are not listed in the table. ^b^ Mental Oxide Semiconductor.

**Table 3:** Confusion matrix of single sensor ^a^.

Classifier	Odor	TGS2620					TGS2603					TGS2600				
		Ethanol	Meat	Cigarette	Sen.	Spe.	Ethanol	Meat	Cigarette	Sen.	Spe.	Ethanol	Meat	Cigarette	Sen.	Spe.
SVM	Ethanol	14,950	2	48	0.9867	0.9934	15,000	0	0	0.9333	0.9667	14901	10	89	0.9888	0.9944
	Meat	0	14,900	100			0	14,802	198			0	15,000	0		
	Cigarette	0	448	14,552			0	2803	12,197			0	405	14,595		
kNN	Ethanol	14,880	70	50	0.8733	0.9367	14,892	52	56	0.9600	0.9800	14,700	201	99	0.9767	0.9884
	Meat	0	10,202	4798			0	13,500	1500			15	14,605	380		
	Cigarette	0	783	14,217			0	192	14,808			2	351	14,647		
Softmax	Ethanol	14,885	108	7	0.9533	0.9767	14,900	25	75	0.9333	0.9667	14,933	0	67	0.9867	0.9934
	Meat	3	14,701	296			100	14,799	101			50	14,890	60		
	Cigarette	5	1682	13,313			0	2700	12,300			3	418	14,579		

^a^ Results for 300*2-fold cross validation (2-CV) using single sensor.

**Table 4:** Confusion matrix of sensor combination ^a^.

Classifier	Odor	TGS2620+TGS2603					TGS2603+TGS2600					TGS2620+TGS2600					TGS2620+TGS2603+TGS2600				
		Ethanol	Meat	Cigarette	Sen.	Spe.	Ethanol	Meat	Cigarette	Sen.	Spe.	Ethanol	Meat	Cigarette	Sen.	Spe.	Ethanol	Meat	Cigarette	Sen.	Spe.
SVM	Ethanol	14,739	261	0	0.9833	0.9916	15,000	0	0	0.9976	0.9988	14,812	188	0	0.9888	0.9944	14,712	288	0	0.9888	0.9944
	Meat	0	15,000	0			0	15,000	0			0	15000	0			0	15,000	0		
	Cigarette	0	491	14,509			0	109	14,891			0	315	14,685			0	214	14,786		
kNN	Ethanol	13,821	360	819	0.9467	0.9733	15,000	0	0	0.9933	0.9966	14,800	200	0	0.9867	0.9934	14,799	201	0	0.9933	0.9966
	Meat	0	15,000	0			0	15,000	0			0	15,000	0			0	15,000	0		
	Cigarette	0	1220	13,780			0	302	14,698			0	398	14,602			0	102	14,898		
Softmax	Ethanol	14,298	0	702	0.9733	0.9866	15,000	0	0	0.9856	0.9928	14,461	0	539	0.9856	0.9928	14,575	0	425	0.9856	0.9928
	Meat	0	15,000	0			0	15,000	0			0	15,000	0			0	15,000	0		
	Cigarette	0	500	14,500			0	649	14,351			0	111	14,889			0	224	14,776		

^a^ Results for 300*2-fold cross validation (2-CV) using 4 sensor combination.

**Table 5:** Confusion matrix in an open environment.

Odor	TGS2603+TGS2600		
	Meat (Predicted)	Cigarette (Predicted)	Ethanol
Meat (Label)	9	0	1
Cigarette (Label)	0	4	6
Ethanol (Label)	1	1	8

## References

[1] Zampolli S., Elmi I., Ahmed F., Passini M., Cardinali G., Nicoletti S., Dori L. (2004). An electronic nose based on solid state sensor arrays for low-cost indoor air quality monitoring applications. Sens. Actuators B Chem..

[2] Annesi-Maesano I., Baiz N., Banerjee S., Rudnai P., Rive S., on behalf of the SINPHONIE Group (2013). Indoor Air Quality and Sources in Schools and Related Health Effects. J. Toxicol. Environ. Health Part B.

[3] Chiaramonti D., Rizzo A.M., Spadi A., Prussi M., Riccio G., Martelli F. (2013). Exhaust emissions from liquid fuel micro gas turbine fed with diesel oil, biodiesel and vegetable oil. Appl. Energy.

[4] Okazaki S., Nakagawa H., Asakura S., Shimizu H., Iwamoto I. (2001). A novel method of temperature compensation for a stable combustion-type gas sensor. Sens. Actuators B Chem..

[5] Getino J., Gutierrez J., Ares L., Robla J., Horrillo M., Sayago I., Agapito J. (1996). Integrated sensor array for gas analysis in combustion atmospheres. Sens. Actuators B Chem..

[6] Frank S.H.M., Weimar U. (2001). Rancidity investigation on olive oil: A comparison of multiple headspace analysis using an electronic nose and GC/MS. Electronic Noses and Olfaction 2000: Proceedings of the 7th International Symposium on Olfaction and Electronic Noses, Brighton, UK, 20–24 July 2000.

[7] Di Natale C., Macagnano A., Nardis S., Paolesse R., Falconi C., Proietti E., Siciliano P., Rella R., Taurino A., D’Amico A. (2001). Comparison and integration of arrays of quartz resonators and metal-oxide semiconductor chemoresistors in the quality evaluation of olive oils. Sens. Actuators B Chem..

[8] Boilot P., Hines E., John S., Mitchell J., Lopez F., Gardner J., Llobet E., Hero M., Fink C., Gongora M.A. (2001). Detection of bacteria causing eye infection using a neural network based electronic nose system. Electronic Noses and Olfaction 2000: Proceedings of the 7th International Symposium on Olfaction and Electronic Noses, Brighton, UK, 20–24 July 2000.

[9] Lin Y.J., Guo H.R., Chang Y.H., Kao M.T., Wang H.H., Hong R.I. (2001). Application of the electronic nose for uremia diagnosis. Sens. Actuators B Chem..

[10] Hofhuis W., de Jongste J.C., Merkus P.J.F.M. (2003). Adverse health effects of prenatal and postnatal tobacco smoke exposure on children. Arch. Dis. Child..

[11] U.S. Department of Health and Human Services (2014). The Health Consequences of Smoking—50 Years of Progress: A Report of the Surgeon General.

[12] Zhao W., Bhushan A., Santamaria A.D., Simon M.G., Davis C.E. (2008). Machine learning: A crucial tool for sensor design. Algorithms.

[13] Zhang S., Xie C., Zeng D., Zhang Q., Li H., Bi Z. (2007). A feature extraction method and a sampling system for fast recognition of flammable liquids with a portable E-nose. Sens. Actuators B Chem..

[14] Qi P.F., Meng Q.H., Jing Y.Q., Zeng M., Ma S.G. Rapid detection of Chinese liquors using a portable e-nose based on C-SVM. Proceedings of the 12th World Congress on Intelligent Control and Automation (WCICA).

[15] Roussel S., Forsberg G., Steinmetz V., Grenier P., Bellon-Maurel V. (1998). Optimisation of electronic nose measurements. Part I: Methodology of output feature selection. J. Food Eng..

[16] Gutierrez-Osuna R. (2002). Pattern analysis for machine olfaction: A review. IEEE Sens. J..

[17] Duda R.O., Hart P.E., Stork D.G. (2012). Pattern Classification.

[18] Boser B.E., Guyon I.M., Vapnik V.N. A training algorithm for optimal margin classifiers. Proceedings of the ACM Fifth Annual Workshop on Computational Learning Theory.

[19] Böhning D. (1992). Multinomial logistic regression algorithm. Ann. Inst. Stat. Math..

[20] Luo D., Hosseini H.G., Stewart J.R. Cigarette Brand Identification Using Intelligent Electronic Noses. http://citeseerx.ist.psu.edu/viewdoc/download?doi=10.1.1.510.5013&rep=rep1&type=pdf.

[21] Monroy J.G., Gonzalez-Jimenez J. (2017). Gas classification in motion: An experimental analysis. Sens. Actuators B Chem..

